# Supplementary material for: Sterol Regulation of Development and 20-Hydroxyecdysone Biosynthetic and Signaling Genes in Drosophila melanogaster
Source: Cells. 2023 Jun 28;12(13):1739. doi: 10.3390/cells12131739 (PMC10340181; doi:10.3390/cells12131739)
Supplement: Supplementary file 1 [file cells-12-01739-s001.zip › cells-2373030-supplementary.pdf]

cells

MDPI

Fig. S1

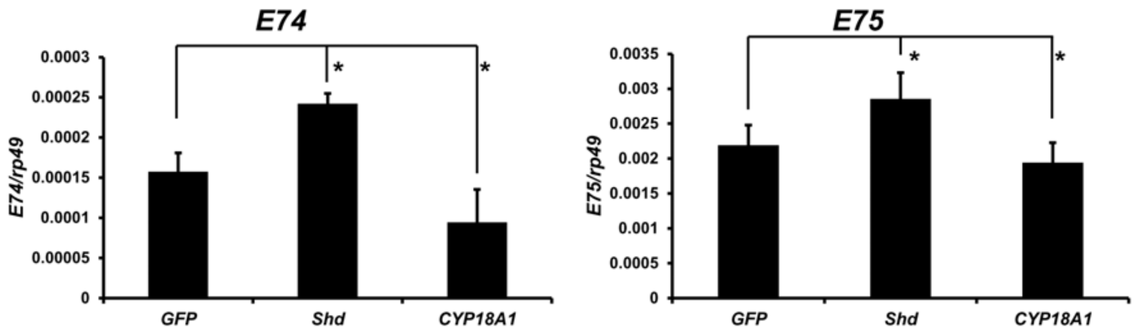

Figure S1. The expression changes in 20E primary response genes under overexpression of Shd and Cyp18a1. Asterisks indicate a significant difference, as calculated using two-tailed unpaired Student's t-test (* $p < 0.05$).

Fig. S2

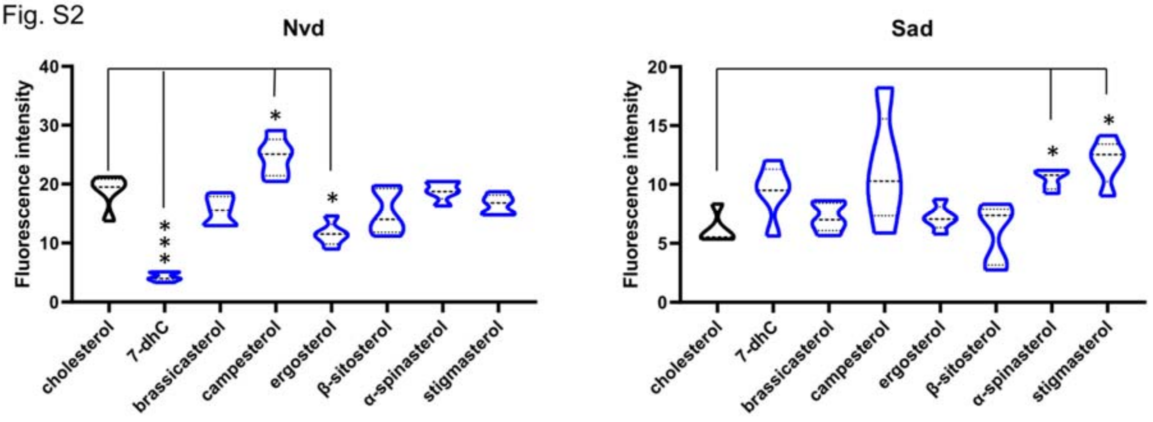

Figure S2. The statistical analyses of relative fluorescence intensities of Nvd and Sad proteins in the prothoracic gland and Shd protein in the fat body. Asterisks indicate a significant difference, as calculated using two-tailed unpaired Student's t-test (* $p < 0.05$; *** $p<0.001$).
